# Transcriptome Analysis and Postprandial Expression of Amino Acid Transporter Genes in the Fast Muscles and Gut of Chinese Perch (*Siniperca chuatsi*)

**DOI:** 10.1371/journal.pone.0159533

**Published:** 2016-07-27

**Authors:** Ping Wu, Yulong Li, Jia Cheng, Lin Chen, Ming Zeng, Yuanan Wu, Jianhua Wang, Jianshe Zhang, Wuying Chu

**Affiliations:** 1 Department of Bioengneering and Environmental Science, Changsha University, Changsha, 410003, China; 2 Collaborative Innovation Center for Efficient and Health Production of Fisheries in Hunan Province, Changde, 415000, China; 3 Institute of Hunan Aquaculture and Fishes, Changsha, 410005, China; University of Maryland, UNITED STATES

## Abstract

The characterization of the expression and regulation of growth-related genes in the muscles of Chinese perch is of great interest to aquaculturists because of the commercial value of the species. The transcriptome annotation of the skeletal muscles is a crucial step in muscle growth-related gene analysis. In this study, we generated 52 504 230 reads of mRNA sequence data from the fast muscles of the Chinese perch by using Solexa/Illumina RNA-seq. Twenty-one amino acid transporter genes were annotated by searching protein and gene ontology databases, and postprandial changes in their transcript abundance were assayed after administering a single satiating meal to Chinese perch juveniles (body mass, approximately 100 g), following fasting for 1 week. The gut content of the Chinese perch increased significantly after 1 h and remained high for 6 h following the meal and emptied within 48–96 h. Expression of eight amino acid transporter genes was assayed in the fast muscles through quantitative real-time polymerase chain reaction at 0, 1, 3, 6, 12, 24, 48, and 96 h. Among the genes, five transporter transcripts were markedly up-regulated within 1 h of refeeding, indicating that they may be potential candidate genes involved in the rapid-response signaling system regulating fish myotomal muscle growth. These genes display coordinated regulation favoring the resumption of myogenesis responding to feeding.

## Introduction

Expression of mRNAs is sensitive to changes in the nutrient status of the skeletal muscles in humans during fasting and insulin infusion or after a high-glycemic meal [1,2]. In fish, recent molecular tools facilitate identifying nutritionally regulated genes related to muscle growth [3]. Such genes might play a role in the stimulation of myogenesis during the skeletal muscles differentiation and development [4,5,6]. Valente et al (2012) reported that nutrient restriction could increase the release of amino acids from muscle fibers and they are used by hepatocytes as the main gluconeogenic precursors in carnivorous fish [7]. During refeeding, it accelerated the amino acids turnover and increased protein synthesis [8]. In the last decade, a considerable amount of effort has been focused on identifying amino acids, particularly leucine, that play a role in stimulating protein synthesis by activating the target of rapamycin complex 1(mTORC1) in the mammals [9]. However, scarce data are available describing the relationship between amino acid transport mechanisms and the fasting–refeeding nutritional status, particularly in the skeletal muscles of aquaculture species.

Amino acid transporters are belonging to the members of the solute-linked carrier (SLC) family, which is ubiquitously expressed in the plasma membrane of many cell types, including the skeletal muscles [10,11]. Until now, 23 amino acid transporters have been identified in Na^+^-dependent systems (A, ASC, B^0^, BETA, Gly, IMINO, N, Nm, Nb, PHE, PROT, APC, and X^C−^) and Na^+^-independent systems (L, T, imino, PAT, asc, X^−AG^, y^+^, y+L, B^0,+^, b^0,+^) [12]. System L includes a heterodimeric complex comprising an L-type amino acid transporter (i.e., neutral amino acids transporter small subunit 2 [LAT2]) and a glycoprotein (solute carrier family 3 member 2 [CD98]), and is responsible for the transport of large neutral amino acids such as leucine [13]. System A comprises sodium-coupled neutral amino acid transporters (e.g., amino acid transporter 2 [ATA2]). A previous study revealed that two transporters, LAT2–CD98 and ATA2, were highly expressed in muscle tissues and had a potential role in promoting muscle growth [14]. Further investigation has confirmed that the protein complex LAT2–CD98 and ATA2 cooperatively activates mTORC1 by increasing the intracellular leucine concentration, whereas the inhibition of ATA2 and CD98 could reduce mTORC1 activity and protein synthesis[15,16,17]. System y+L is composed of two subunits, a polytopic membrane protein (i.e., Y+L amino acid transporter 1 (y+LAT1) or Y+L amino acid transporter 2 [y+LAT2]) and an associated type II membrane protein (4F2 heavy chain) [18]. Recently, the y+LAT1 system has gained increasing attention because it transports large amounts of cationic and neutral amino acids and provides essential nutrients for animal growth and the energy required for metabolism and reproduction [19,20]. Therefore, further investigation and characterization of amino acid transporters in fish will provide a more comprehensive understanding of the regulation of skeletal muscle growth and may be crucial in improving aquaculture applications.

Fasting–refeeding protocols are commonly used as the model system to investigate the regulation of muscle growth in teleosts[21]. The protocols include a relative long time of fasting and then continuous refeeding, by which the transcript abundance was assayed over several days or weeks [22,23]. By contrast, a single satiating meal treatment was well designed to study the transcriptional responses to nutrient availability, with relatively high temporal resolution [7]. Gut tissue is an important organ in nutritional digestion and absorption of vertebrate animals [24]. The amino acid transporters play an important role in the amino acid absorption in the gut [25]. The latest research shows skeletal muscle is not only the most abundant tissue in the fish body mass but also plays a large role in whole-body metabolism [21]. The expression levels of mRNAs are sensitive to changes in nutrient status of the skeletal muscle [7]. Amino acids are involved in the regulation of major metabolic pathway and considered as signaling molecules in muscle and gut tissues. Therefore, we chose these two tissues for the detection of amino acid transporter gene expression. However, until now, scare study has been carried out to assay amino acid transporter expression in teleost skeletal muscles, especially for aquaculture species during the postprandial period. *Sinperca chuatsi* is one of the most commercially valuable carnivorous fish species in aquaculture in China and Eastern Asia [26,27]. Its high nutritional value, high protein content, and appealing taste stimulate its large-scale culture in China [28]. In the present study, we analyzed transcriptome data from the fast muscles of Chinese perch to determine the number and expression level of amino acid transporters in the skeletal muscles of this species. Furthermore, the effect of a fasting–refeeding treatment on the amino acid transporter response involved muscle growth was investigated. These fundamental studies provide insights into increasing the muscle mass in fish production.

## Materials and Methods

### Ethics Statement

This study was conducted in accordance with the recommendations in the Guide for the Care and Use of Laboratory Animals of the National Institutes of Health. The protocol was approved by the Institutional Animal Care and Use Committee (IACUC) of Changsha University (permit #20128945–1). All surgeries were performed under sodium pentobarbital or tricaine methanesulfonate (MS-222) anesthesia, and every effort was made to minimize the animal suffering. All fish-handling procedures during the studies were approved by the Institutional Animal Care and Use Committee (IACUC) of Changsha University.

### Experimental Conditions and Sampling

Chinese perch (*Siniperca chuatsi*) were reared under standard conditions at the Hunan Fisheries Science Institute (Changsha, Hunan, China). The five 1-year-old fishes were euthanized by immersion in water containing 2g L^-1^ sodium pentobarbital. Fast muscle samples were collected from five 1-year-old fish and subjected to transcriptome sequencing. Two similar sizes of Chinese perch juveniles (average body mass, 100 g) were reared in duplicate net cages (5 × 5 × 2 m), with 50 fish per tank. The fish were provided a live feed (mud carp *Cirrhinus molitorella*; average body weight, 10 g) during 3-week experiments (body weight, 1.5%). The fish were firstly fasted for 1 week, and then all of the fish were fed a single meal to visual satiation. Furthermore, the fish were sampled at 0 h and at 1, 3, 6, 12, 24, 48, and 96 h after the meal, with 5 fish sampled at each time point. The 5 fishes were selected at each sampling point and were euthanized by immersion in water containing 0.2 g·L^−1^ MS-222. The intestine and stomach contents of each fish were determined and photographed. The fast skeletal muscles were then collected from the dorsal myotome. All samples were kept in liquid nitrogen −80°C until further processing.

### Transcriptome Sequencing

Total RNA was extracted from the fast muscles of adult Chinese perch by using the RNA extraction KitII(Takara Biotechnology, Dalian, China) and beads with oligo (dT) were used to isolate poly(A) mRNA. Fragmentation buffer was added to disrupt the mRNA to produce short fragments and they were then used as templates. A random hexamer primer was used to synthesize the first-strand cDNA. Second-strand cDNA was synthesized using a buffer containing dNTPs, RNaseH, and DNA polymerase I. The short fragments were then purified using the QiaQuick PCR extraction kit(Takara Biotechnology, Dalian, China) and resolved with an elution buffer (EB buffer) for end reparation and by adding poly(A). Further, the short fragments were connected with sequencing adapters. After agarose gel electrophoresis, suitable fragments were selected as templates for polymerase chain reaction (PCR) amplification. Finally, the library was sequenced using Illumina HiSeq^™^ 2000.

Sequencing was conducted at the Beijing Genomics Institute and transcriptome de novo assembly was performed using the short reads assembly program Trinity [29]. Trinity was first combined to reads with a certain length of overlap to form longer fragments or contigs. The reads were then mapped back to the contigs. Paired-end reads can detect contigs from the same transcript as well as the distances between these contigs[30]. Finally, Trinity was connected to the contigs, and sequences that could not be extended on either end were prepared. Such sequences were defined as unigenes. Unigene sequences were first aligned by using BLASTX to search protein databases, such as NR, SwissProt, Kyoto Encyclopedia of Genes and Genomes (KEGG), and COG (e < 0.00001), and then aligned by using BLASTN to search the nucleotide database NT (e < 0.00001).

### Fasting–Refeeding Experiment

Before the experiment we chosen GeNorm to analyze the stability of transcription of reference genes including Glyceraldehyde-3-phosphate dehydrogenase (GAPDH), *β*-actin, 18 S rRNA gene, hypoxanthine phosphoribosyltransferase 1-like (HPRT1), epinephelus coioides ribosomal protein S29 (RPS29) and ribosomal protein L13 (RPL13). GeNorm Analysis revealed that *β*-actin (geNorm stability value M = 0.23) was the most stable genes in different tissues. The expression levels of eight amino acid transporter genes were quantitativelly assayed using real-time PCR (qRT-PCR) with beta-actin as the internal control. Primers for the selected genes were designed according to the transcriptome sequencing results (Table 1). Total RNAs from the two fasting–refeeding experiments as previously described were extracted using the TRIzol^R^ reagent (Invitrogen, Beijing,China) and then treated with RNAse-free DNAse I (Promega, USA) in the presence of an RNAse inhibitor (Sigma, Shanghai, China), followed by ethanol precipitation. The obtained RNA was reverse-transcribed using SuperScript III RNase H-reverse transcriptase (Invitrogen, Beijing, China) according to the manufacturer’s instructions. Negative controls contained no cDNA template.

**Table 1:** Primers used for quantitative RT-PCR.

Forward	Sequence(5′-3′)	Reverse	Sequence(5′-3′)
ATA4-F	TTGCTCCACACCTTCACCAA	ATA4-R	CTGAACTCTCGGCCACTGAAC
CD98-F	CTAGGCTGGGTAGGCATGCT	CD98-R	GTCCAACCTCGCCTCAACAC
PAT1-F	ACAGCCATCTTTGCCTTCGA	PAT1-R	CAGCAGTTTGGCAGGTTGAG
ATA2-F	CCACTGAGCGGCCAATACTC	ATA2-R	GGCCACGAGGAGAATCACAA
CAT2-F	CGGCATTCTCGTGGGTGTAC	CAT2-R	TGTGAAGCATGCGTGTTAGTGTC
EAAT1-F	GCAGGGAAGATTGTGGAGATG	EAAT1-R	CCACACGGTTGTTCTCCTCCA
LAT2-F	GGCTATCAAACCCACATCATACTCC	LAT2-R	CTGCTGACATGGGTGAACTGCT
y+LAT2-F	CTGGCTACTGAACGACGGGG	y+LAT2-R	GCCTGTTCTTAGTCATCGTCCC
*β*-actin-F	CTTGACTTCGAGCAGGAG	*β*-actin-R	GGCATACAGGTCTTTACGG
RPL13-F	CACAAGAAGGAGAAGGCTCGGGT	RPL13-R	TTTGGCTCTCTTGGCACGGAT
HPRT1-F	CATACCAAAGCATTACGCAGAAG	HPRT1-R	CACCTCGAATCCTACAAAGTCCG
RPS29-F	TCACCCCAGAAAATTCGGACAGG	RPS29-R	GTATTTACGGATCAGACCGTGTC
18 S -F	GGAATGAGCGTATCCTAAACCC	18 S -R	CTCCCGAGATCCAACTACAAGC
GAPDH-F	ATCAAGGAAGCGGTGAAGAAGG	GAPDH-R	CGAAGATGGAGGAGTGGGTGTC

cDNA samples from the gut and skeletal muscles were used as templates for quantitative RT-PCR assays with the SYBR Green PCR reaction kit (Stratagene, Shanghai, China), and its amplification reaction was performed using the Stratagene Mx3005 system (Stratagene, CA, USA). The reaction mix includes 2 μL of each cDNA template, 2.5 μL of SYBR Green mix, 1 μL of each gene specific primer (10 μmol L^−1^, Table 1), and 8.5 μL of nuclease-free water to a 25 μL of final reaction volume. The following amplification protocol was adopted: (i) predenaturation at 95°C for 60 s; (ii) amplification and quantification, repeated 40 cycles of 95°C for 5 s and 60°C for 25 s; and (iii) melting curve program (65°C–95°C with a heating rate of 0.1°C/s and fluorescence measurement). Each product was identified through dideoxy-mediated chain termination sequencing at the BioSune Biotech Company (Shanghai, China). The relative expression ratio (*R*) of the target mRNA was calculated by *R* = 2^−ΔΔ*Ct*^, where *Ct* is the cycle threshold. The basic equation used was ΔΔ*Ct* = (*Ct*_target gene_−*Ct*_housekeeping gene_)_experiment_−(*Ct*_target gene_−*Ct*_housekeeping gene_)_control_.

The mRNA expression levels were then analyzed through one-way analysis of variance and regression analysis with SPSS (SPSS, USA IBM). Duncan’s multiple range tests were used to compare the differences between the control and experimental groups. The differences were considered statistically significant when P < 0.05. Data are shown as mean ± standard error (n = 6).

## Results

### Transcriptome Assembly Annotation

**Fig 1:**
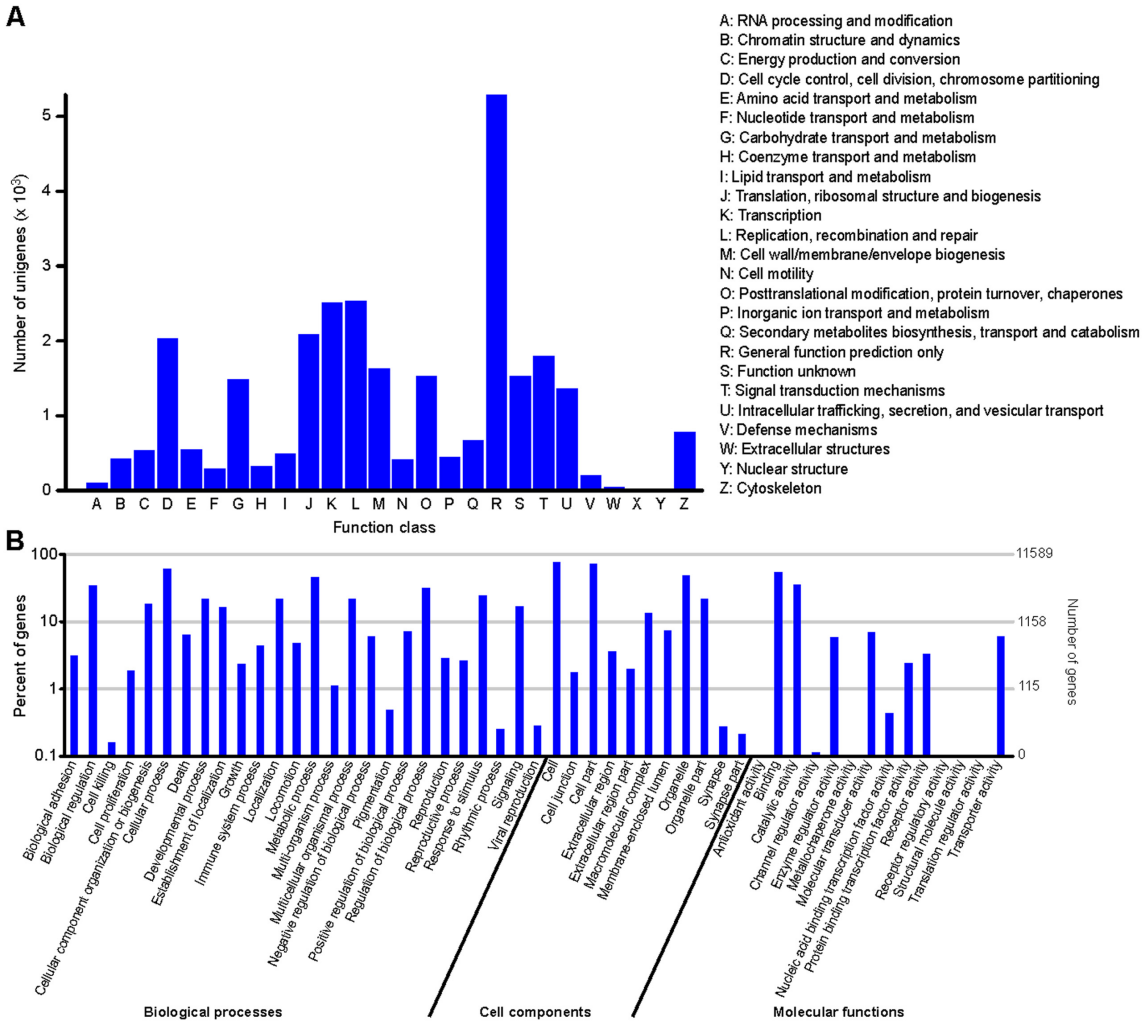
COG classification (A) and GO classification (B).

**Fig 2:**
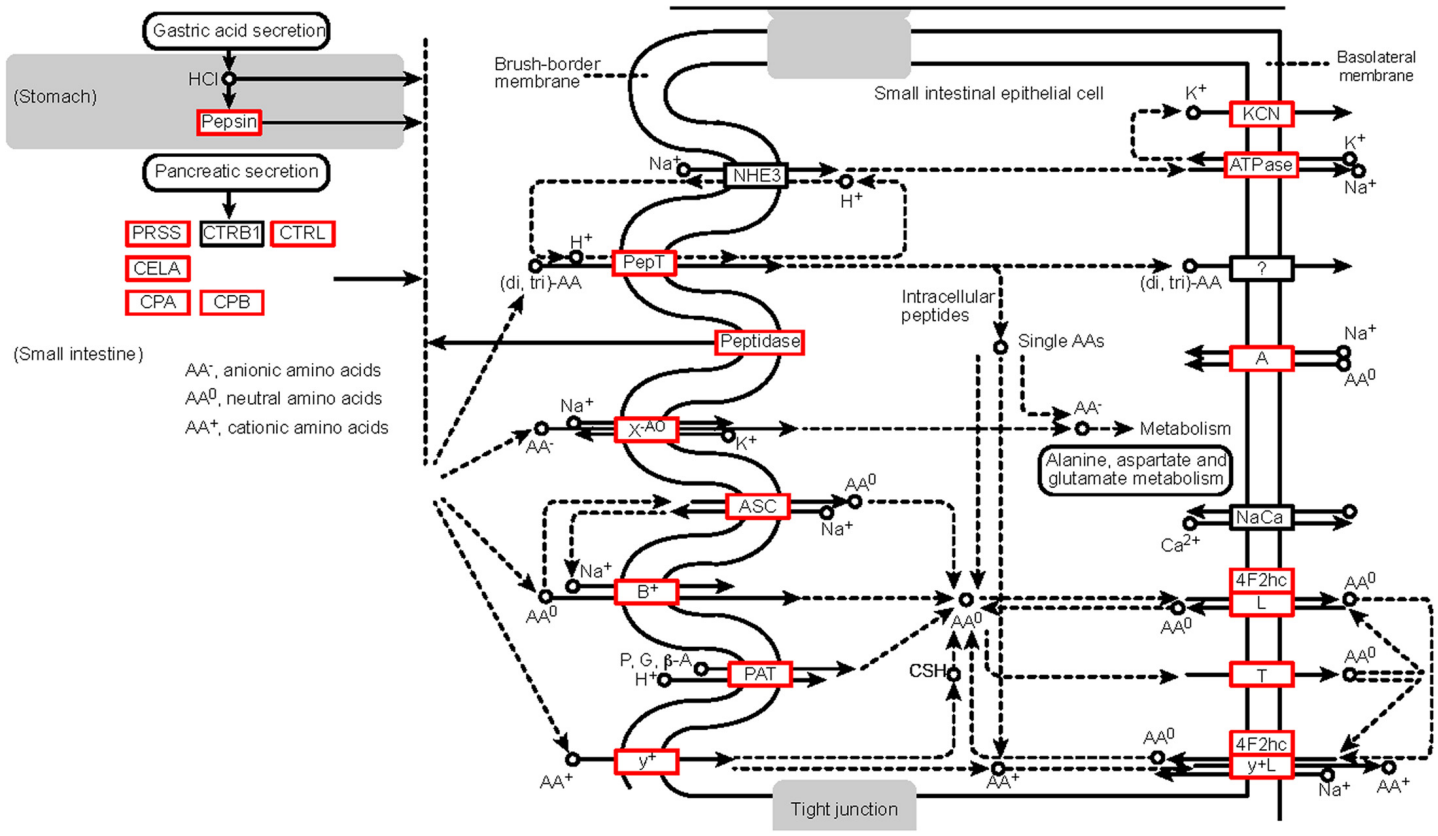
Twenty-three amino acid transport systems identified in animals and nine in the muscles of Chinese perch. Eight Na^+^-independent transporters and 10 Na^+^-dependent were identified. The amino acids transporter genes found in muscle were marked with a red box to indicate the difference between the intestinal and muscle tissues.

For the transcriptome analysis, one library was constructed from the mixed pool of muscle mRNA from five individuals. The complete clean reads for the library have been uploaded to the NCBI Sequence Read Archive site (http://www.ncbi.nlm.nih.gov/sra/), accession nos SRX1738860. The total number of the annotated isotigs and unigenes was 156,749 and 75,534, respectively (Table 2). Unigenes were annotated using the NR, NT, SwissProt, KEGG, COG and GO databases. In the COG function classification of Unigenes sequence (Fig 1), 25 classifications are associated with various functions. However, more than half the functions are only general function predictors. The GO classification (Fig 1) contains the biological process, cellular component, and molecular function. We also observed 241 related metabolic pathways. The number of unigenes annotated with each database was then counted. Nine families of transporters with 47 members are related to the transport of amino acids and oligopeptides in fish [12]. By summing the reported literature, we found that the amino acid transporter genes research is concentrated in the gut. Twenty-three amino acid transport systems identified in the intestinal carrier pathway were showed in the Fig 2. The latest research shows the. amino acids are involved in the major metabolic regulation in muscle. In this paper, twenty-one amino acid transporter genes were identified by searching protein and gene ontology databases from the muscle transcriptome library of Chinese perch. Six families of amino acids and oligopeptide transporters, namely SLC1, SLC3, SLC7, SLC17, SLC36, and SLC38, were observed in the fast muscles of the Chinese perch. Among these, eight transporters were Na^+^-independent and 10 were Na^+^-dependent (Fig 2). SLC1, with 13 members found in teleost fish so far (six members were found in this study), transports high-affinity glutamate and neutral amino acids. SLC3, with two members (one member was found), encodes the heavy subunits of the heteromeric amino acid transporters. SLC7, with eight members (four members were found), belongs to cationic amino acid–glycoprotein associated transporters. SLC17, with seven members (three members were found), is involved in the vesicular storage of the glutamate and the glycoprotein degradation and metabolism. SLC36, with one member, which was also observed in this study, is involved in H^+^-coupled amino acid transport. SLC38, with eight members (three members were found in this study), functions as a Na^+^-coupled neutral amino acid transporter.

**Table 2:** List of the currently known Amino acid transporters families in teleost fish and mandarin fish in the fast muscle. HUGO: The Human Genome Organisation; Total: total number of members in each family.

HUGO	Family	Total	mandarin fish
SLC1	High-affinity glutamate and neutral amino acid transporter family	13	6
SLC3	Heavy subunits of the heteromeric amino acid transporters	2	1
SLC7	Cationic amino acid transporter/ glycoprotein-associated family	8	4
SLC15	Proton oligopeptide cotransporter family	3	1
SLC17	Vesicular glutamate transporter family	7	3
SLC32	Vesicular inhibitory amino acid transporter family	1	1
SLC36	Proton-coupled amino acid transporter family	1	1
SLC38	System A and system N sodium-coupled neutral amino acid transporter family	8	3
SLC43	Na+-independent, system-L-like amino acid transporter family	4	1

### Feeding Response during the Single Meal Treatment

**Fig 3:**
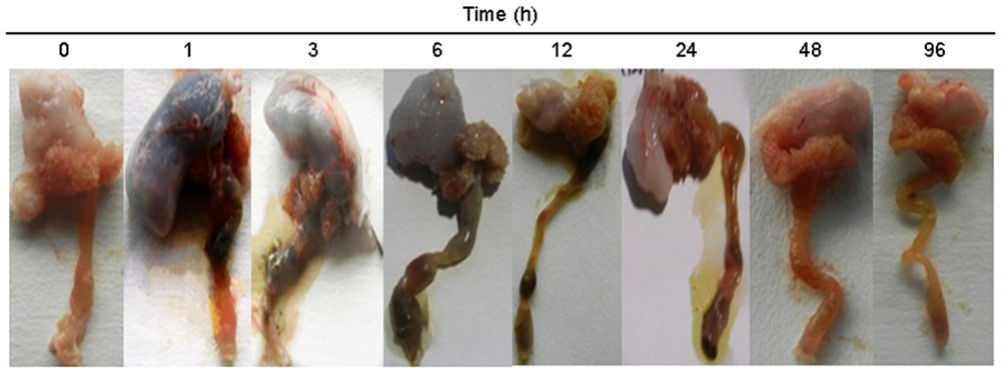
The gut was completely filled at 1–6 h after feeding and emptied within 48–96 h. We investigated postprandial changes in juvenile Chinese perch following a single satiating meal (body mass, approximately 100 g) following fasting for 1 week.

The fish mass and length ranged between 100 and 125 g and 23 and 27 cm, respectively, and were similar at all sampling points (P < 0.05). The gut content of the Chinese perch increased significantly after 1 h and remained high up-to 6 h following the meal (Fig 3). The fish were kept hungry after the meal, and their stomachs were nearly emptied after 48–96 h. These results confirm that all the sampled fish had ingested food, and their intestines were completely emptied between 48 and 96 h after the meal treatment.

### The Eight Amino Acid Transport Genes

**Fig 4:**
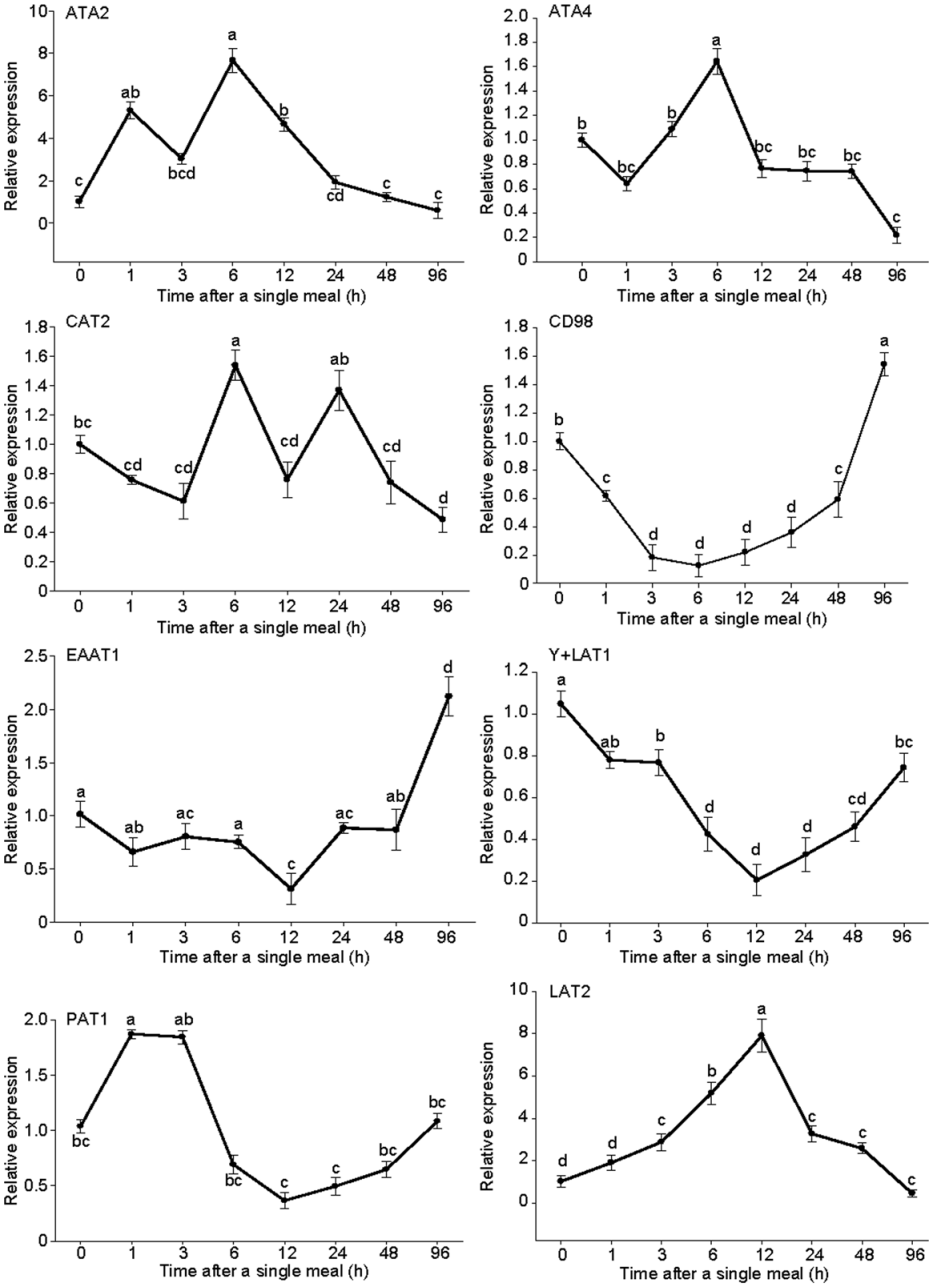
Expression of eight amino acid transporters in fast skeletal muscles after a single meal: A) ATA2, B) ATA4, C) CAT2, D) CD98, E) EAAT1, F) y+LAT1, G) PAT1, and H) LAT2. The values are expressed as the mean ± SEM (n = 6) of the normalized transcript levels of each amino acid transporter. Statistical difference between time points is indicated by different letter notations (P < 0.05).

**Fig 5:**
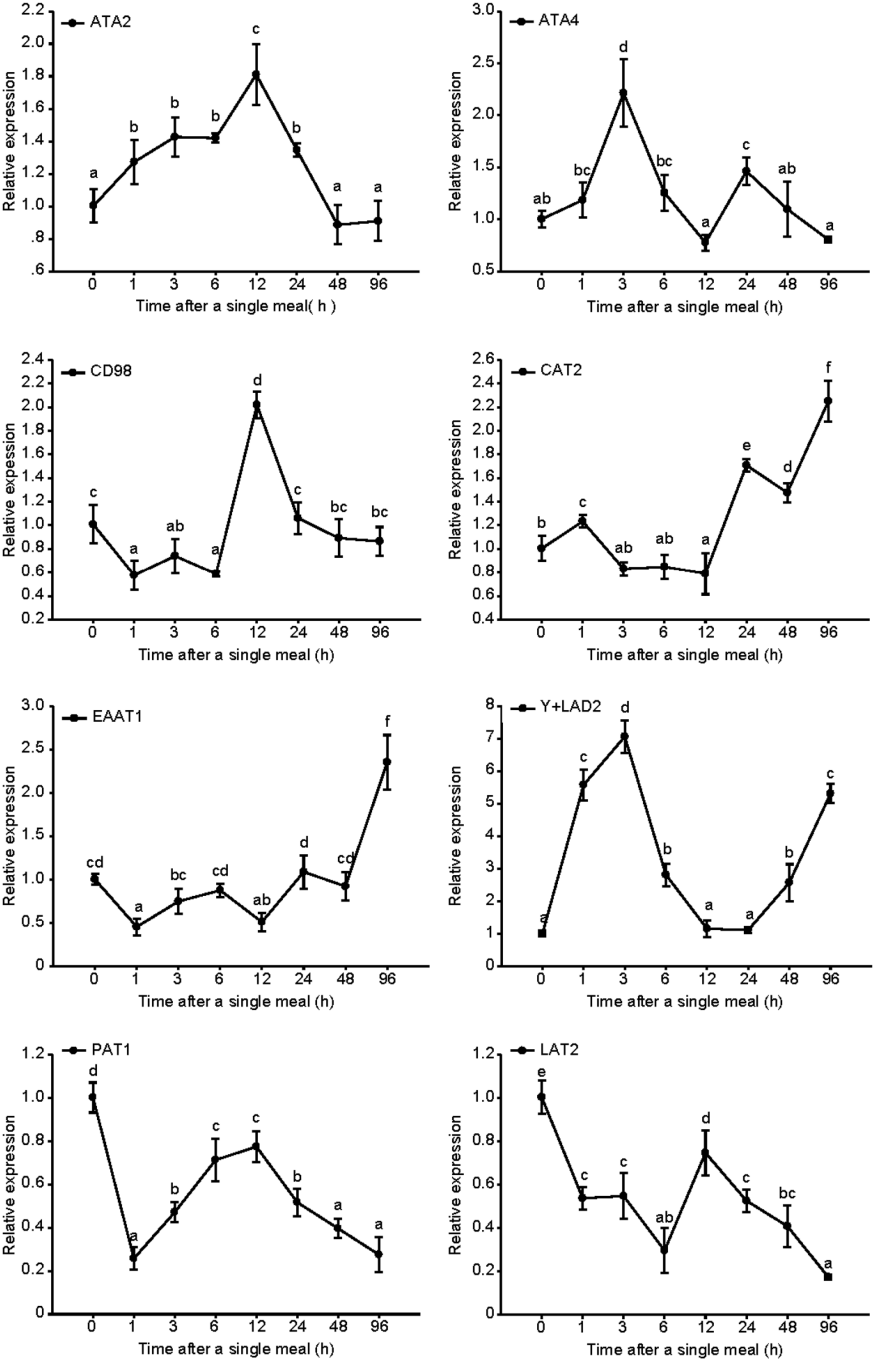
Expression of eight amino acid transporters in the gut after a single meal: A) ATA2, B) ATA4, C) CAT2, D) CD98, E) EAAT1, F) y+LAT1, G) PAT1, and H) LAT2. The values are expressed as the mean ± SEM (n = 6) of the normalized transcript levels of each amino acid transporter. Statistical difference between time points is indicated by different letter notations (P < 0.05).

To investigate the segmental expression of the eight nutritionally regulated genes for 1 week, the fish were re-feeding a meal after fasting for one week. We selected eight genes belonging to six amino acid transport systems, and compared the mRNA levels of the eight amino acid transporters in mandarin fish at eight time points (0, 1, 3, 6, 12, 24, 48, and 96 h following the single meal) through quantitative RT-PCR. In the fast skeletal muscles (Fig 4), as an LAT, which includes a heterodimeric complex, the expression of LAT2 and the glycoprotein CD98 was high compared with the control group as well as ATA2, which belongs to System A with the sodium-coupled neutral amino acid transporters. At 1 h following the single meal, ATA2 and a H^+^-coupled amino acid transporter (putative anion transporter, proton-coupled amino acid transporter 1[PAT1]) increased significantly, whereas CD98 decreased. The remaining transporters showed no significant difference compared with the control group. At 6 h, as the gut content began emptying, low affinity cationic amino acid transporter 2 (CAT2), neutral amino acid transporter 4 (ATA4), and ATA2 reached their highest expression levels, whereas CD98 showed the lowest expression level. Compared with the levels at 6 h, at 48 h when the gut content was being emptied, the expression levels of CAT2, ATA4, ATA2, and LAT2 decreased significantly; however, the expression level of CD98 and y+LAT1 increased significantly. Unlike in the muscles, in which no transporter changed significantly at 1 h following the single meal (except for ATA4), other transporters in the intestines changed significantly (Fig 5). Meanwhile, the relative expression of CD98, excitatory amino acid transporter 1 (EAAT1), and PAT1 was the lowest. At 3 h, ATA4 and y+LAT1 continued showing an increasing trend and reached their highest expression level. In gastric emptying, the relative expression levels of some transfer vectors (ATA2, CD98, and LAT2) increased considerably, whereas those of other transfer vectors (ATA4, y+LAT1, and EAAT1) decreased. At 12 h following the single meal, all transporter levels started fluctuating. Within 48 h after gastric emptying, CAT2, EAAT1, and y+LAT1 continued increasing, at which point CAT2 and EAAT1 reached their highest level.

## Discussion

### Transcriptome Analysis of the Amino Acid Transporter Family

The SoLute Carrier (SLC) gene family comprise of most of membrane transporters and they play roles in transporting substrates across membranes [31]. At present, representatives of 50 SLC families have been identified in teleost fish, although no member has been identified in the SLC28 family to date. The SLC series in fish has at least 338 putatively functional protein coding genes, whereas that in humans comprise 51 families and at least 378 genes including passive transporters, ion-coupled transporters, and exchangers [12,32]. Up to date, various transporters of the SLC series have been cloned from different fish species. In a survey, zebrafish (*Danio rerio*) represented the most relevant species with 304 genes, whereas others, such as Atlantic salmon (*Salmo salar*), rainbow trout (*Oncorhynchus mykiss*), fugu (*Takifugu rubripes*), mefugu (*Takifugu obscurus*), and Japanese eel (*Anguilla japonica*), were reported to have 53, 22, 16, 11, and 10 genes, respectively [12]. In total, 232 SLC genes were observed in our transcriptome in Chinese perch. Amino acid transporters are membrane proteins mediating the transport of amino acids across the plasma membrane, and they play a crucial role in protein synthesis and absorption. Furthermore, 23 amino acid transport systems have been identified in animals; in our study, nine systems were identified in the fast muscles of Chinese perch. The Chinese perch transcriptome contains six SLC1 genes, whereas that of zebrafish contains 13 SLC1 genes. In addition, only seven members of the slc1 gene family have been described in humans and mice, and nearly half the genes are present in zebrafish (Table 2). The differences in the transport gene members reflect specificity in fish species and protein synthesis.

### Expression of Cationic Amino Acid Transporters

Numerous studies have described the transition of the nutrient-sensing pathway from a catabolic to anabolic state in the skeletal muscles [3,4,5,33]. The mechanisms involved in the regulation of metabolism depend on the crosstalk between nutritional ingestion and transportion. Amino acids are the important factor in the regulation of nutritional ingestion and adsorption. Postprandial plasma free amino acid levels increased significantly in trout 2 h after the meal. FAA levels remained significantly elevated 8 h after refeeding. In these fish, plasma levels of total FAAs decreased significantly lower than levels measured in fasted fish 24 h after feeding[34]. The gut content of the Chinese perch increased significantly after 1 h and remained high for 6 h and emptied within 48–96 h following the meal. According to the FAA levels and the ingestion time after the meal, it is sufficient to study the postprandial expressional pattern of the amino acid transporter genes in the gut and muscle of Chinese fish during 96 hours after refeeding.

**Fig 6:**
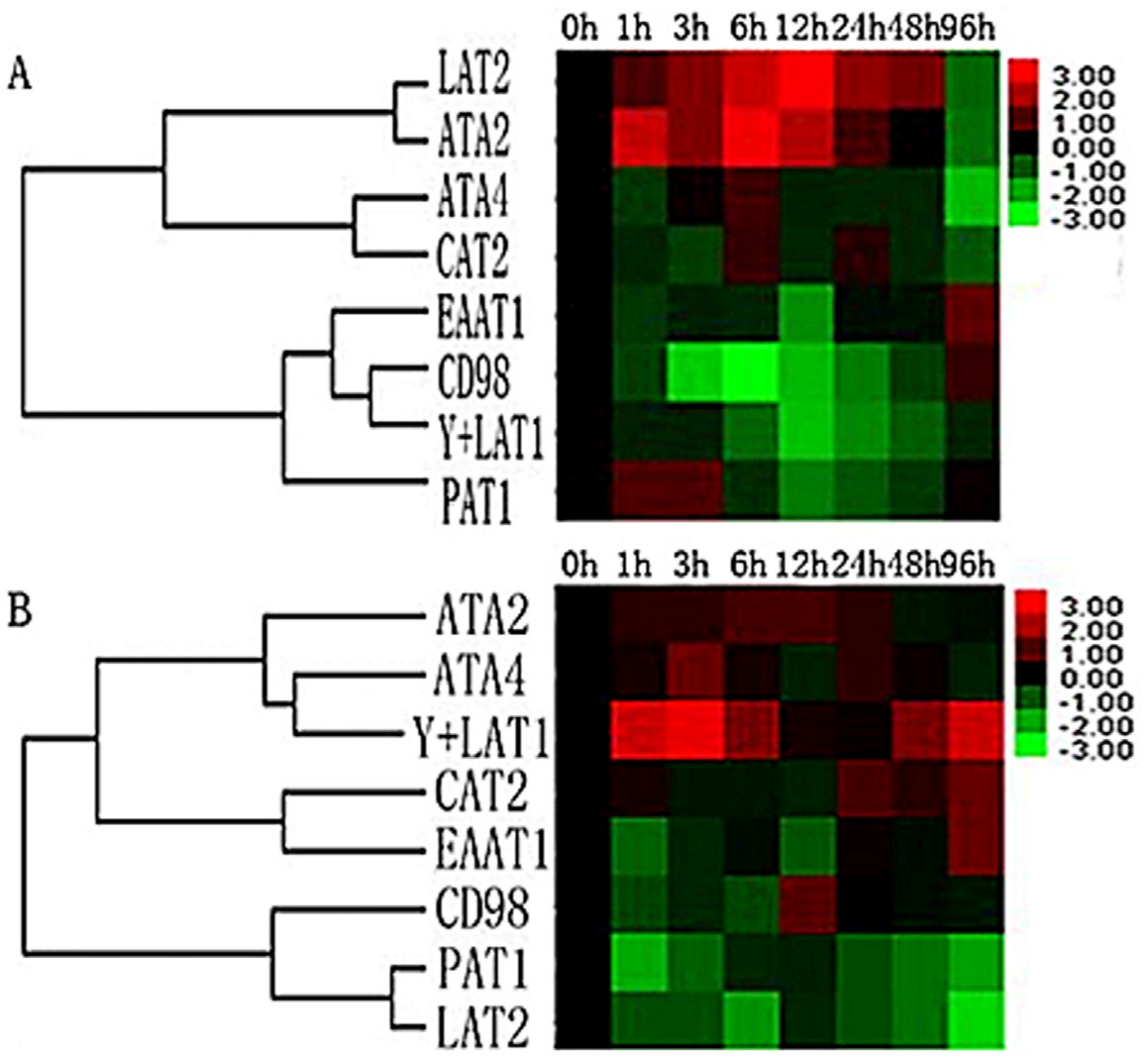
Heat map summary and unsupervised hierarchical clustering analysis of the genes in the muscles (A) and gut (B) according to the similarity in their expression across different postprandial times (0–96 h).

Upon to now, studies on fasting and refeeding following the assays of transcript abundance have been reported in many species, such as Atlantic salmon [22,35], rainbow trout [36,37], Atlantic halibut [23], sea bass [38], and sea bream [39]. Studies on insulin-like growth factor (IGF) signalling stimulating transcriptional changes in zebrafish [40] and Atlantic salmon [41] were also reported. Earlier studies demonstrated that amino acids are essential for tissue protein synthesis and other metabolic functions [41,42]; the amino acids could be transported by different transporters [43,44,45]. Cationic amino acid transporters (CATs) are widely distributed in different tissues and play a crucial role in the transport of arginine, histidine, lysine, and ornithine, which involve in regulating homeostasis [46,47]. Therefore, understanding the expression of CATs is essential. In our study, two neutral transporters (ATA2 and ATA4) and an acid transporter (EAAT1) showed the same change trend in the muscles and gut. However, an alkaline transporter (CAT2) and two other neutral transporters (y+LAT1 and LAT2) showed obvious changes (Fig 6).

Lysine and arginine are typically the first limiting amino acids in diets [48,49]. The polytopic membrane protein y+LAT1 transports large amounts of cationic and neutral amino acids to provide essential nutrients for animal growth and the energy metabolism [19,20]. This protein is a membrane protein glycosylation complex; however, its expression requires a type II membrane protein glycosylation complex, 4F2hc (also CD98hc), which is a combination of heavy chains. Only when combined with 4F2hc, LAT1 exerts transfer activity [43,50,51,52]. Wang et al. (2009) reported that the degradation of lysine and other basic amino acids in the small intestine play a role in modulating the intestinal growth and mucosal thickness [53] because their metabolites are essential for DNA and protein synthesis [54,55]. In fish and other vertebrates, nutritional status could influence the gene expression levels of amino acid transporters. As previously reported, when European sea bass was fasted from 3 days to 21 days, the mRNA expression levels of peptide transporter 1 (PEPT1) in the upper and lower intestinal sections reached to the highest level at the 7th day [56]. In rats, the PepT1 expression level increased apparently from 5th day of fasting[57]. However, the expression of y+LAT1 in grass carp showed a different response model after fasting. Studies by Yang et al (2014) demonstrated that the y+LAT1 mRNA levels in the foregut and midgut of grass carp were different after 1 day of fasting[29]. Higher levels of y+LAT1 mRNA expression were observed in the intestines, suggesting that it is a crucial part in controlling the transportation of short chains of amino acids present in the diet in the epithelium of the small intestinal brush border membrane [29]. In our study, y+LAT1 expression level of increased considerably at 6 h after the single meal. Our findings support the notion that an appropriate balance among amino acids (including both nutritionally essential and nonessential amino acids) in diets is crucial for maximizing the efficiency of animal production[58]. Therefore, understanding the expression of the y+LAT1 transporter is crucial.

System Y has four amino acid transporters: CAT1, CAT2A and CAT2B, CAT3, and CAT4. CAT1–CAT3 have a 60% homology, but CAT4 has only approximately 40% homology [59]. CAT1 is one of the main systems of y transporters, expressed in nearly all tissues and cells. CAT1 is mainly responsible for the transport of three types of amino acids: L-lysine, arginine, and ornithine [60]. Therefore, the CAT family of alkaline amino acids plays a major role during the transfer process. The present findings accord with those in a study by Luı´sa [7], who reported that a single meal affects the expression of several growth-related genes. Another studies have indicated the importance of CAT in the small intestine, specifically in maintaining the homeostasis of basic amino acids and overall protein nutrition in the body [51]. In the present study, CAT2 mRNA levels continued to increase in the gut and increased in the muscle with the emptying of the stomach; however, the levels decreased after gastric emptying. This is possibly because CAT2 can utilize the chemical potential of the cytoplasmic membrane coupling union that is assembled to transfer the substrate [61]. This observation requires further investigation.

## Conclusions

This is the first study on the transcriptome annotation of the skeletal muscles of Chinese perch in a muscle growth-related gene analysis. In this study, we generated mRNA sequence data from the fast muscles of Chinese perch by using Solexa/Illumina RNA-seq. Twenty-one amino acid transporter genes were annotated by searching protein and gene ontology databases, and postprandial changes in their transcript abundance were assayed following a single satiating meal in juvenile of Chinese perch. Our results reveal that upon the satiating treatment, five transporter transcripts were markedly up-regulated within 1 h of refeeding, indicating that they may be promising candidate genes involved in a rapid-response signaling system that regulates fish myotomal muscle growth. These genes display coordinated regulation in favoring the resumption of myogenesis as an early response to feeding. Therefore, the present findings provide a crucial foundation for a more comprehensive understanding of the physiological function of amino acid transporters and their nutritional regulation, which may also provide practical applications in aquaculture.
